# Effect of Platelet-Rich Plasma Injection on Mild or Moderate Carpal Tunnel Syndrome: An Updated Systematic Review and Meta-Analysis of Randomized Controlled Trials

**DOI:** 10.1155/2020/5089378

**Published:** 2020-11-14

**Authors:** Chunke Dong, Yan Sun, Yingna Qi, Yuting Zhu, Hongyu Wei, Di Wu, Chungen Li

**Affiliations:** ^1^Department of Orthopaedics and Traumatology, Beijing Hospital of Traditional Chinese Medicine, Capital Medical University, No. 23, Meishuguan Houjie (Art Gallery Back Street), Dongcheng District, Beijing 100010, China; ^2^Beijing University of Chinese Medicine, No. 11, North Third Ring East Road, Chaoyang District, Beijing 100029, China

## Abstract

**Objective:**

To evaluate efficacy of platelet-rich plasma (PRP) injection in carpal tunnel syndrome (CTS), we conducted this meta-analysis, as well as proposed a protocol for its application in curative processes.

**Methods:**

All randomized controlled trials (RCTs) of PRP for the management of mild or moderate CTS were included in this study. Database search was conducted from study inception to July 2020, including PubMed, Embase, Web of Science, and Cochrane Library. We used visual analogue scores (VAS) and the Boston Carpal Tunnel Questionnaire (BCTQ) as evaluation tools for primary outcomes. Second outcomes comprised cross-sectional area (*Δ*CSA) and electrophysiological indexes including distal motor latency (DML), sensory peak latency (SPL), motor nerve conduction velocity (MNCV), sensory nerve conduction velocity (SNCV), compound muscle action potential (CMAP), and sensory nerve action potential (SNAP). The pooled data were analyzed using RevMan 5.3. Subgroup and sensitivity analyses were conducted with the evidence of heterogeneity. Egger' test was used to investigate publication bias.

**Results:**

9 RCTs were finally screened out with 434 patients included. Control groups comprised corticosteroid injection in 5 trials, saline injection in 1 trial, and splint in 3 trials. At the 1st month after follow-up, only *Δ*CSA between the PRP group and the control group showed significant difference (*P* < 0.05). In the 3rd month, there were statistically significant differences in VAS, BCTQ, SPL, SNCV, and *Δ*CSA between two groups (*P* < 0.05), while no statistically significant differences were found in the remaining outcomes. In the 6th month, there were statistically significant differences at BCTQ (*P* < 0.05) in primary outcomes and *Δ*CSA (*P* < 0.05) in secondary outcomes between two groups. As to adverse events in PRP injection, only one study reported increased pain sensation within 48 h after injections.

**Conclusion:**

This systematic review and meta-analysis demonstrates that the PRP could be effective for mild to moderate CTS and superior to traditional conservative treatments in improving pain and function and reducing the swelling of the median nerve for a mid-long-term effect. To some extent, the electrophysiological indexes also improved after PRP injection compared with others conservative treatments.

## 1. Introduction

Carpal tunnel syndrome (CTS) is one of the most disturbing entrapment neuropathy in upper limbs [1] affecting up to 5% of the adult population [2]. Due to the pathogenesis of the median nerve (MN) compression, which passes through the carpal tunnel, CTS is characterized by representative symptoms such as paresthesia and pain in areas innervated by the MN. More seriously, motor deficit or atrophy of the innervated muscles emerges in severe CTS [3] which strikes a huge impact on quality of life.

CTS therapies usually include conservative and surgical management. Surgical therapy is best documented scientifically, but it is not without flaws; therefore, in mild and moderate CTS, conservative therapy is often preferred [4, 5]. More recently, Wolny and Linek [6, 7] assessed the effectiveness of neurodynamic techniques in conservative therapy of CTS, but the conclusions are not definitive. A community-based cohort revealed that approximately 60% to 70% of patients who underwent conservative treatment did not have symptom relief at the 18-month follow-up, which indicated limited long-term efficacy [8]. Corticosteroids have been utilized for perineural injection as early as 1980 and achieved a definite effect including CTS, although a Cochrane Review stated that corticosteroid injections only provided symptomatic benefit at 1-month follow-up. Moreover, steroid application may be associated with adverse events, e.g., neurotoxicity and degenerative tendon rupture [9, 10]. As for patients with severe CTS, surgical treatment became a desired trial [1, 11]; however, the decision should be cautious in view of the failure rate in surgery ranging 7-75% as reported [12]. Thus, it is necessary to seek a novel noninvasive and cost-effective conservative treatment.

Since 2014, platelet-rich plasma (PRP) has gradually emerged in neuropathy, with admissible success rates [13, 14]. PRP is an autologous blood product collected and centrifuged from the patient's blood and comprises a high concentration of platelets. In addition, several high concentrations of growth factors are believed to play crucial roles in tissue regeneration and healing. When PRP is injected to patients themselves, the aforementioned ingredients promote wound healing and angiogenesis and improves axonal regeneration in the entrapment area. Recently, the profit regarding nerve fiber regeneration was also demonstrated in an animal study [15]. Nevertheless, long-term clinical outcome of PRP remains unknown. What is more, it is reported that the concentrations less than 4 to 6 times or higher than 8 times may be ineffective or conversely inhibit the healing process [16, 17]. Indeed, the argument did exist about the centrifugation technique and the enrichment percentages of blood [18–20].

So far, several RCTs compared PRP injection to varieties of other conservative treatments with different assessments. To provide better evidence of efficacy and safety of PRP injection in CTS, we conducted this meta-analysis, as well as proposed a protocol for its application in curative processes.

## 2. Materials and Methods

The present meta-analysis was conducted based on the Preferred Reporting Items for Systematic Reviews and Meta-Analyses (PRISMA) statement [21]. The protocol for our systematic review was registered on INPLASY (2020100077) and available from doi: 10.37766/inplasy2020.10.0077.

### 2.1. Criteria for including Studies

All RCTs of PRP injection for the management of more than 10 participants per group with mild or moderate CTS were included in this study. The primary outcomes will be assessment of pain symptom using the VAS and BCTQ [22, 23], which was designed definitely for CTS. BCTQ contains 2 distinct scales, the Symptom Severity Scale (BCTQs) and the Functional Status Scale (BCTQf). Secondary outcomes involved *Δ*CSA and clinical results of nerve electrophysiology related to motor and sensory nerves. Articles that reported at least one outcome were included.

### 2.2. Criteria for excluding Studies

Articles without the outcome measures of interest were excluded. Non-RCTs, retrospective studies, cross-sectional studies, animal studies, in vitro biomechanical studies, case reports, comments, letters, editorials, and reviews were excluded.

### 2.3. Database Searches

Electronic databases including PubMed, Embase, Cochrane Library, and Web of Science were searched up to July 2020 for RCTs involving PRP in the management of mild or moderate CTS. The search strategy for PubMed was as follows: The keywords for the study object (MeSH words or free words) included (“Carpal Tunnel Syndrome” OR “Carpal Tunnel Syndromes” OR “Syndrome, Carpal Tunnel” OR “Syndromes, Carpal Tunnel” OR “Amyotrophy, Thenar, Of Carpal Origin” OR “Median Neuropathy, Carpal Tunnel” OR “Compression Neuropathy, Carpal Tunnel” OR “Entrapment Neuropathy, Carpal Tunnel”). For the intervention strategy, the keywords were “Platelet-Rich Plasma” OR “Plasma, Platelet-Rich” OR “ Platelet Rich Plasma “. For the study design strategy, the keywords were “Randomized Controlled Trial” OR “Randomized” OR “Placebo”. We only included English-language articles. In addition, the reference lists of selected articles and relevant reviews were manually searched for any additional trials.

### 2.4. Data Extraction and Quality Assessment

The results were managed with Endnote X7 software, and duplicate studies were automatically deleted. Next, two authors (CK.D. and Y.S.) independently reviewed all titles and abstracts related to the eligibility criteria described above. The full text of the literature was reviewed thoroughly for a final inclusion. All disagreements were resolved by reaching a consensus with the third author (YN.Q.).

Data were extracted by two authors (CK.D. and YN.Q.) from selected studies independently using a standardized form. Information for each eligible study included author information, publication year, method of randomization and blinding, data sources, sample sizes, demographic database, parameters of concentration and centrifugation, detailed interventions, treatment course, outcomes, follow-up duration, and adverse events. When a 100-point NRS score was used, it was converted to a 10-point VAS score [24]. Data in median, interquartile range, and mean ± 95% confidence interval (95% CI) were converted to mean ± standarddeviation (SD) according to the Cochrane Handbook [25]. We extracted data by manual measurements from the published figures when not reported numerically. If necessary, we contacted the relevant authors in trials for more original data.

Two authors (YT.Z. and HY.W.) independently assessed the risk of bias of the included studies based on the Cochrane Collaboration's tool according to six items: random sequence generation, allocation concealment, blinding of participants and personnel, incomplete outcome data, selective reporting, and other biases. Disagreement was resolved by the third author (D.W.).

### 2.5. Statistical Analysis

Meta-analysis was performed using software RevMan 5.3. Continuous data were expressed as mean ± SD and calculated through the mean difference (MD) or standardized mean difference (SMD) with 95% CI. Heterogeneity across included studies was assessed with the Cochran *Q* test (the level of significance was set at 0.1) [26]. The *I*^2^ score was also used to determine the degree of heterogeneity (*I*^2^ < 50%, no obvious heterogeneity; *I*^2^ > 50%, large or extreme heterogeneity) [26]. A random-effect model was used for heterogeneous statistical data. Otherwise, a fixed-effect model was performed. Sensitive analysis or subgroup analysis was used to investigate the source of heterogeneity. Meta-analysis results were also assessed using forest plots, and *P* < 0.05 was considered statistically significant.

## 3. Results

### 3.1. Literature Search

**Figure 1:**
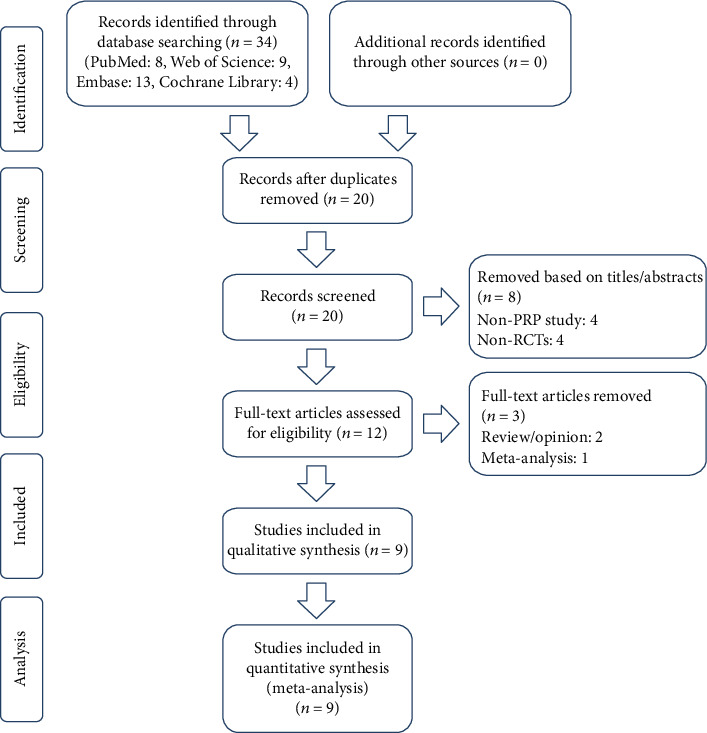
Flow diagram of the study selection process for the meta-analysis.

In total, 34 citations were identified after an initial systematic search. Afterwards, we reviewed abstracts and titles of included studies, selected the relevant information, and removed duplication independently, and 20 studies were selected. Finally, 9 RCTs were screened out after reading the full text (Figure 1).

### 3.2. Characteristics and Risk of Bias of Included Studies

**Figure 2:**
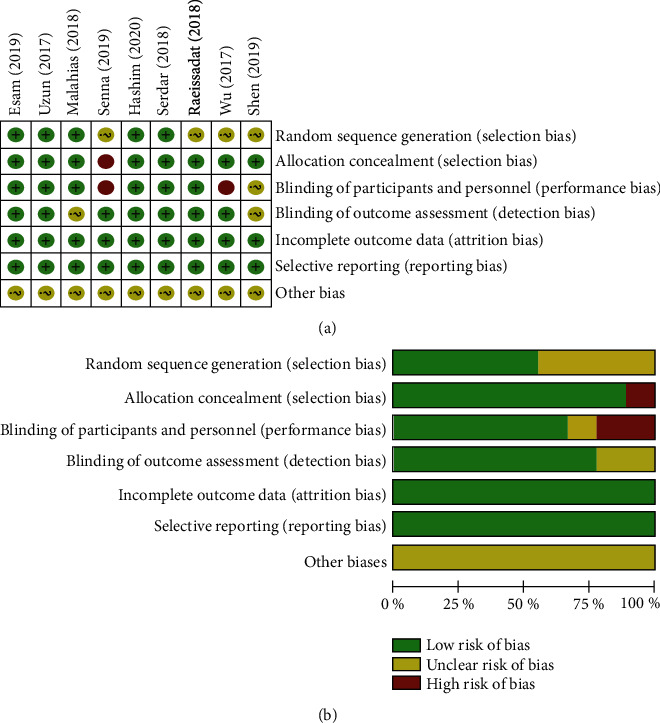
The methodological quality of the included studies. Risk of bias summary (a) and risk of bias graph (b): +: low risk of bias; −: high risk of bias; ?: bias unclear.

A total of 9 RCTs published with 434 patients were finally included in this meta-analysis. Characteristics of all studies are shown in Table 1. All studies compared clinical outcomes of PRP injection versus other conservative treatments for management of mild to moderate CTS. Besides, control groups comprised corticosteroid injection in 5 trials [13, 27–30], saline injection in 1 trial [31], and a splint in 3 trials [32–34]. Of the 9 included studies, 7 studies were considered to have a low risk of bias, while the 2 remaining studies were found to have a high risk of bias. Random sequence generation was found in 5 studies. Allocation concealment was found in 8 studies, and blinding of participants and personnel were found in 6 studies. Blinding of outcome assessment was found in 7 studies. As shown in Figure 2, incomplete outcome data and selective reports were not found in 9 studies.

### 3.3. Comparative Analysis of PRP and Other Conservative Therapies

After carefully reading and summarizing the included RCTs, we used VAS and BCTQ as evaluation tools for primary outcomes. Second outcomes comprised *Δ*CSA [35] and electrophysiological indexes including DML, SPL, MCV, SNCV, CMAP, and SNAP. Meanwhile, we conducted a subgroup analysis at different follow-up times (at 1, 3, and 6 months). Besides, the difference of MD and SD (*Δ*CSA and SNCV) between baseline and follow-up was compared in the subgroup analysis for *Δ*CSA and SNCV.

#### 3.3.1. First Month after Follow-Up

**Figure 3:**
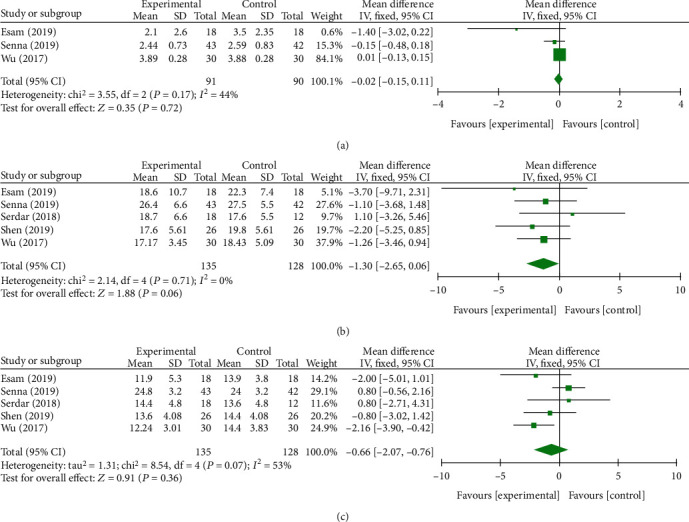
Forest plot of primary outcomes at 1 month of follow-up. They are VAS (a), BCTQs (b), and BCTQf (c).

**Figure 4:**
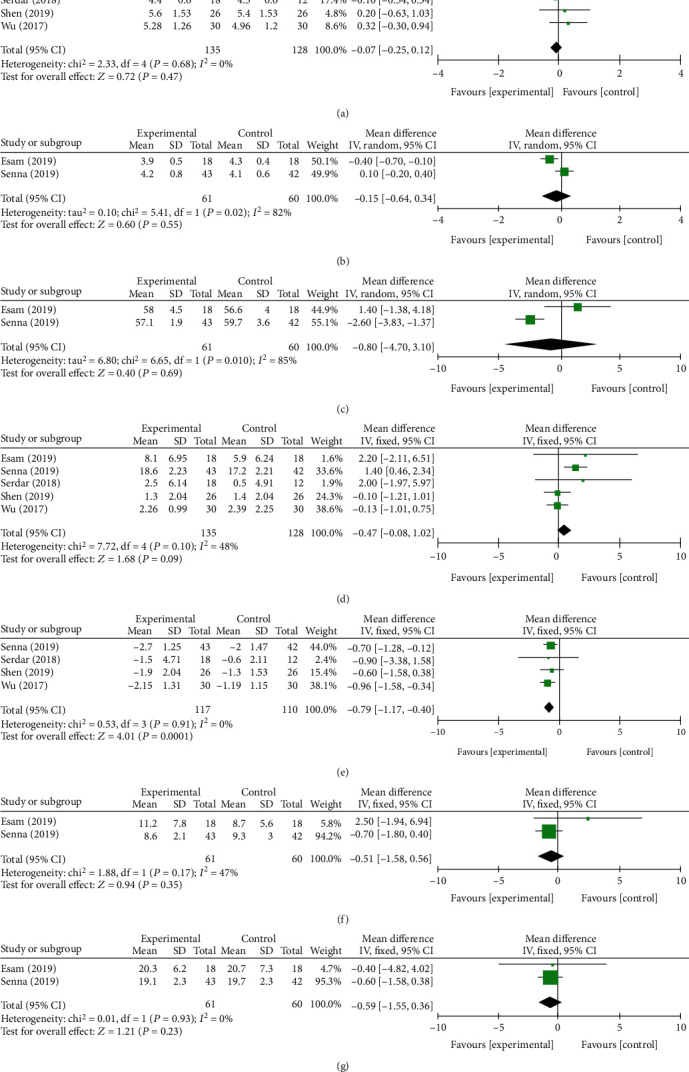
Forest plot of secondary outcome at 1 month of follow-up. They are DML (a), SPL (b), MNCV(c), SNCV (d), *Δ*CSA (e), CMAP (f), and SNAP (g).

At the first month after follow-up, a total of five component studies [27–29, 33, 34], including 707 and 1237 subjects, provided data on primary outcomes and secondary outcomes, respectively. As reported in Figures 3 and 4, only *Δ*CSA between the PRP group and the control group showed significant difference (*P* < 0.0001, Figure 4(e)) with no additional significance of heterogeneity. However, there were no significant differences in remaining outcomes (*P* > 0.05).

#### 3.3.2. Three Months after Follow-Up

**Figure 5:**
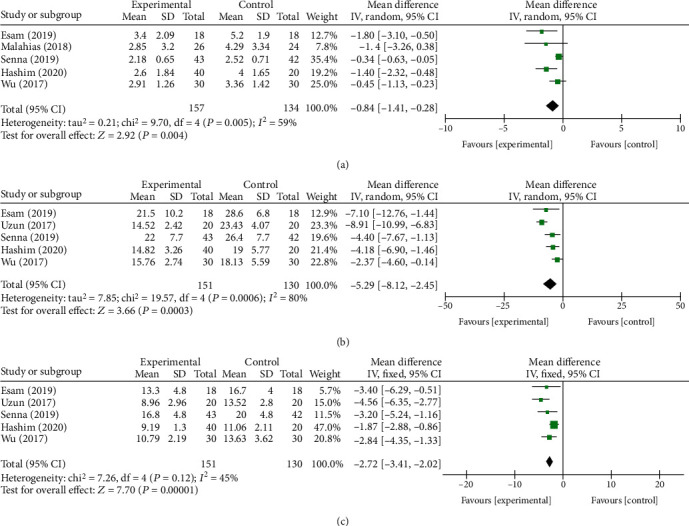
Forest plot of primary outcomes at 3 months of follow-up. They are VAS (a), BCTQs (b), and BCTQf (c).

**Figure 6:**
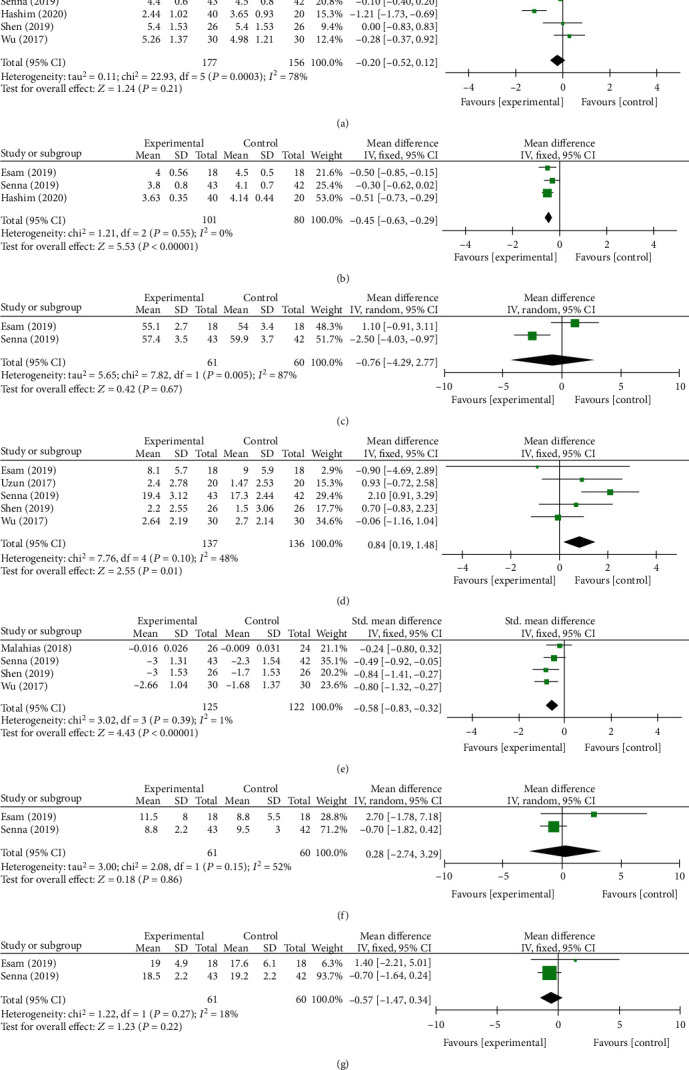
Forest plot of secondary outcomes at 3 months of follow-up. They are DML (a), SPL (b), MNCV(c), SNCV (d), *Δ*CSA (e), CMAP (f), and SNAP (g).

In the 3rd month, a total of 907 and 1347 subjects provided data on primary outcomes [13, 28–31, 33] and secondary outcomes [13, 27–31, 33], respectively. There were statistically significant difference at VAS, BCTQs, BCTQf, SPL, SNCV, and *Δ*CSA between two groups (*P* = 0.004, Figure 5(a); *P* = 0.0003, Figure 5(b); *P* < 0.00001, Figure 5(c); *P* < 0.00001, Figure 6(b); *P* = 0.01, Figure 6(d); and *P* < 0.00001, Figure 6(e)). No statistically significant differences were found in the remaining indexes. The fixed-effect model was preformed when *I*^2^ < 50%. Otherwise, a random-effect model was performed. We conducted a subgroup analysis based on different control treatment methods, and the SNCV during the 3-month follow-up compared to corticosteroid injection was statistically different (*P* = 0.01).

#### 3.3.3. Six Months after Follow-Up

**Figure 7:**
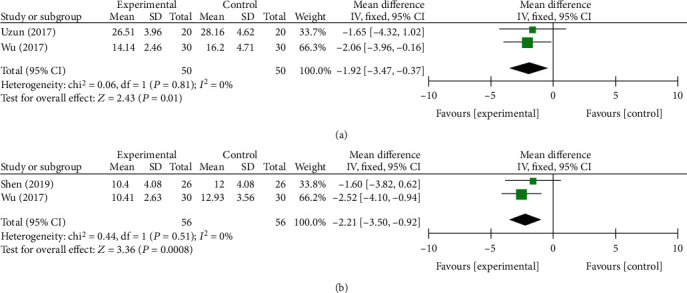
Forest plot of primary outcomes at 6 months of follow-up. They are BCTQs (a) and BCTQf (b).

**Figure 8:**
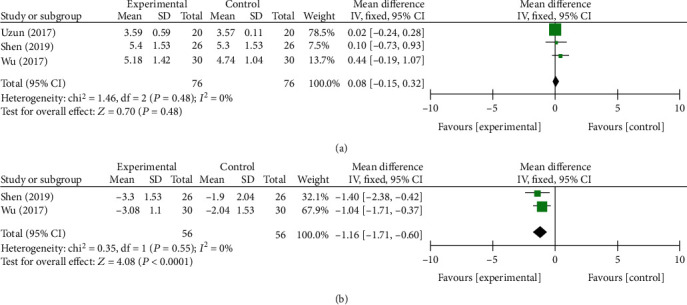
Forest plot of secondary outcomes at 6 months of follow-up. They are DML (a) and *Δ*CSA (b).

In the 6th month, a total of three component studies [13, 27, 33], including 304 and 264 subjects, provided data on primary outcomes and secondary outcomes, respectively. There were statistically significant differences at BCTQs (*P* = 0.01, Figure 7(a)) and BCTQf (*P* = 0.0008, Figure 7(b)) in primary outcomes and *Δ*CSA (*P* < 0.0001, Figure 8(b)) in secondary outcomes between two groups. On the contrary, no statistically significant differences were found in DML (*P* = 0.48, Figure 8(a)). The fixed-effect model was preformed due to no heterogeneity.

### 3.4. Publication Bias

**Figure 9:**
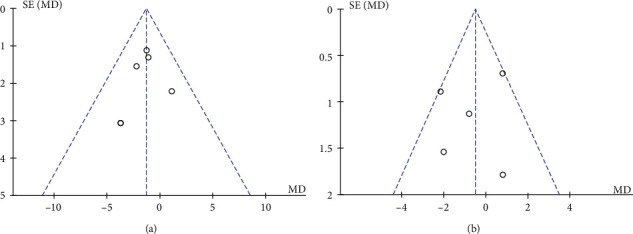
Funnel plot to detect publication bias: 1-month BCTQs (a) and BCTQf (b). MD: mean difference; SE: standard error.

**Figure 10:**
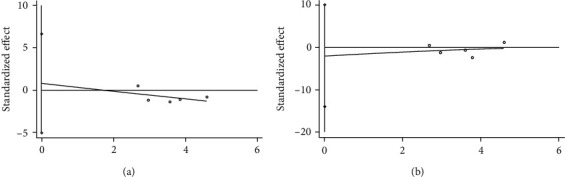
Egger's publication bias plot: 1-month BCTQs (a) and BCTQf (b).

Funnel plots of 1-month BCTQs and BCTQf were generated for evaluation of publication bias because both were the main indicators and had enough relevant studies. As observed, the data indicated moderate publication bias by uncertainty (Figure 9). Afterwards, Egger's test was conducted and showed no evidence of publication bias for 1-month BCTQs (*P* = 0.703, Figure 10(a)) and 1-month BCTQf (*P* = 0.635, Figure 10(b)).

### 3.5. Sensitivity Analysis

Sensitivity analysis was performed by omitting 1 study in each turn to investigate the influence of a single study on the overall outcome. 1-month BCTQf showed substantive difference compared to the original analysis when removing the study of Senna et al. (*P* = 0.02, *I*^2^ = 0%). When Uzun et al.'s study [13] was removed, BCTQs at 3rd-month follow-up was *P* < 0.00001, *I*^2^ = 1% without additional heterogeneity. Similarly, there was no heterogeneity in DML after removing Hashim et al.'s study [30] (*P* = 0.91, *I*^2^ = 0%). Besides, the results did not show substantive difference compared to the original analysis in remaining indicators.

## 4. Discussion

As a disturbing entrapment neuropathy with a high incidence, current treatments have shown limitations, and more attempts at conservative treatment for CTS are needed to effectively relieve symptoms and avoid disease progression and surgery. In recent years, PRP has aroused wide concern for efficacy and safety on neurosurgery and orthopedics [36, 37], and PRP injection was gradually used for management of CTS. Previous meta-analysis by Catapano et al. [38] concluded promising but confounded results limited by the small number of studies available (4 studies), short-term follow-up, and high heterogeneity. Therefore, selection bias and completeness of outcome data may affect reliability of results. In the present analysis, 9 high-level studies were included. In addition, comprehensive nerve function indicators relating to motor and sensory were pooled and calculated under the same follow-up month. Thus, we aimed to further evaluate efficacy and safety of PRP injection in CTS, as well as proposing a protocol for its application in curative process.

In the last years, PRP was clinically applied for eliminating pain in muscle injuries and chronic neuropathic pain. Moreover, Kuffler [39] stated that PRP may take effect by eliminating inflammation and initiating a series of biological processes such as tissue remodeling, wound repair, and axonal regeneration. In this analysis, we found promising outcomes in BCTQ at 3 and 6 mo follow-up and VAS at 3 mo follow-up in the PRP group. The BCTQs evaluated symptoms of patients with CTS broadly which comprised the frequency, duration, and severity of tingling, numbness, and pain. As such, the aforementioned result demonstrated the more effective relief of symptoms in long-term follow-up in PRP injection compared with other conservative treatments. In most of the included studies, corticosteroid injections were used, and hormones were consistently considered to have short-term effects on pain relief. This also explains why there was no difference between the two groups during the 1 mo follow-up. Therefore, PRP injection was also effective in reducing pain symptoms in the short term. Moreover, PRP injection showed potential long-term benefits in nerve function as subgroup analysis based on different control treatment methods showed that the SNCV value during the 3-month follow-up with PRP injection was statistically different compared to that with corticosteroid injection (*P* = 0.01).

Generally, CTS for mild to moderate is reversible; thus, intervention at this stage is critical which means treatments should focus on not only relief of symptoms but also enhancing the regeneration of nerve tissue and completely eliminate the causes of compression. Indeed, PRP has a higher concentration of growth factors after centrifugation such as insulin-like growth factor, transforming growth factor, fibroblast growth factor, platelet-derived growth factor, and vascular endothelial growth factor [40–42], which were closely associated to regeneration of nerve tissue. Moreover, evidence was addressed in animal studies. A rat study by Park and Kwon [43] compared dextrose and PRP injection with the saline injection, and the results of *Δ*CSA and DML were positive. In the present meta-analysis, PRP had a lasting effect on *Δ*CSA compared to the control group during whole follow-up period which indicated that PRP injection reduced the swelling of the flexor tenosynovitis. According to Allampallam et al. [44], the flexor retinaculum of individuals with carpal tunnel syndrome is physiologically altered and growth factors resulted in higher mitogenic response, stimulation of type III collagen production, and more alpha 2 than alpha 1 collagen production which were closely associated in pathological changes beneficial for CTS. On the other hand, several authors revealed that platelet counts might not be the best predictor of platelet biological activity, and platelet counts were not always proportional to the number of growth factors [45–47]. However, enlargement of the median nerve can be caused by a variety of changes, such as inflammation, fibrosis, intima or axonal edema, and demyelination or remyelin [1]. Thus, the underlying mechanism has not been understood, and more studies are needed in the future. Overall, the present study demonstrated the promising effect of PRP injection on CTS.

Notably, there were significant differences in sensory electrophysiological examination (SPL: *P* < 0.00001; SNCV: *P* = 0.01) at the 3rd-month follow-up between the PRP injection and control groups which was in accordance with relief of symptoms. Several authors reported that there may be difference between symptoms and electrophysiological result, as shown in our sensitivity analysis, partly due to large myelinated fibers responsible for electrophysiological examinations instead of small sensory fibers which are related to symptoms of CTS [48]. Nevertheless, our results shed a light on neurological function improvement which deserved persistent studies. However, motor nerve function did not improve according to MNCV and DML. Indeed, previous studies showed that the electrodiagnostic measurement had limitations in predicting the curative outcome for conservative treatments [49, 50]. Furthermore, nerve recovery was a rather slow process which might last up to 18 months, and longer follow-up may be needed in the future to address the issue considering the positive result in BCTQf (3 month: *P* < 0.00001; 6 months: *P* = 0.0008).

As to adverse events in PRP injection, only one study by Senna et al. [28] reported increased pain sensation within 48 h after injection. Afterwards, patients got symptom relief by receiving paracetamol and local ice application. Complications were uncommon as surgeons were experienced and ultrasound guidance was widely applied to avoid neurovascular damage. Of the included studies, 6 out of 9 (66.7%) used ultrasound guidance. More importantly, the blood sample came from the patients themselves, and antibody response was avoided. Above all, PRP injection was safe and well tolerated.

The appropriate PRP preparation and accurate enrichment percentages that maintain an optimal balance between the advantages and potential side-effects remain controversial. It was reported that the platelets may get prematurely activated by excessive concentrations [36] or pipetting [51], and consequently, growth factors were early released. As mentioned before, the concentrations less than 4 to 6 times or higher than 8 times may be ineffective or conversely inhibit the healing process [16]. Among the included studies, different concentration processes were used resulting in variable concentrations of platelets and other elements. Unfortunately, the enrichment percentages were not reported except for one study by Hashim et al. [30]; however, no significant results were observed between two PRP groups. Hence, further studies are necessary to address this issue.

### 4.1. Limitations

The present study had some limitations. Firstly, included studies might result in selective and performance biases due to the absence of random allocation, allocation concealment, and blinding. Heterogeneity may have been caused by study design, which induced by the different PRP injection doses, and varying intervention methods in the control group. Secondly, all studies were followed up within six months which was relatively short to get information on long-term effects and side effects. Longer follow-up for 1-2 years may be required. Thirdly, all studies are limited to English which may lead to language bias. Fourth is the lack of research on effective studies at different PRP doses, and more studies are needed to address the issue. Finally, given the limited number of the included studies in the analysis, the findings should be confirmed in future research with more relevant RCTs to obtain more reliable and conclusive data.

## 5. Conclusions

Although a similar early effect, our study demonstrates that PRP could be effective for mild to moderate CTS and superior to traditional conservative treatments in improving pain and function and reducing the swelling of the median nerve for a mid-long-term effect. To some extent, the electrophysiological indexes also improved after PRP injection compared with other conservative treatments. Overall, PRP injection was an effective and safe additional therapy for patients with CTS.

## Figures and Tables

**Table 1:** Characteristics of all the trials included in the meta-analysis.

Study	Country	Sample size		Age (years)		Gender (M/F)		Characteristics of CTS	Therapy in intervention group	Therapy in control group
		I	C	I	C	I	C			
Hashim (2020)	Egypt	20	20	48.8 ± 6.62	49.1 ± 6.06	3/17	2/18	Mild to moderate	PRP injection, 1 ml	Methylprednisolone acetate
Shen (2019)	China	20	26	56.8 ± 1.7	58.5 ± 2.1	2/18	1/25	Moderate	PRP injection, 3 ml	5% dextrose injection
Senna (2019)	Egypt	43	42	38.3 ± 6.4	40.7 ± 9.4	8/35	6/36	Mild to moderate	PRP injection, 2 ml	Methylprednisolone acetate
Malahias (2018)	Greece	26	24	60.46 ± 14.39	57.17 ± 16.14	NR		Mild to moderate	PRP injection (ultrasound guided), 2 ml	0.9% normal saline injection
Esam (2019)	Egypt	18	18	38.5 ± 8	36.6 ± 8.8	2/16	2/16	Mild to moderate	PRP injection (ultrasound guided), 2 ml	Methylprednisolone acetate
Uzun (2016)	Turkey	20	20	48.8 ± 5.8	48.5 ± 6.1	4/16	4/16	Mild	PRP injection, 2 ml	Triamcinolone acetonide
Wu (2017)	China	30	30	57.87 ± 1.51	54.27 ± 1.34	3/27	5/25	Mild to moderate	PRP injection (ultrasound guided), 3 ml/night splint	Splint
Serdar (2018)	Turkey	18	12	47.5 (28-63)	50 (31-56)	1/17	1/11	Mild to moderate	PRP injection (ultrasound guided), 1 ml	Splint and acetaminophen
Raeissadat (2018)	Iran	21	20	51.2 ± 9.82	47.23 ± 7.11	0/21	0/20	Mild to moderate	PRP injection, 1 ml/splint	Splint

I: PRP injection group; C: control group; NR: not reported.

## Data Availability

The data used to support the findings of this study are available from the corresponding author upon request.
